# Effect of Simulated Gastrointestinal Conditions on Biofilm Formation by *Salmonella* 1,4,[5],12:i:-

**DOI:** 10.1155/2014/153956

**Published:** 2014-06-30

**Authors:** R. Seixas, M. Gabriel, J. Machado, L. Tavares, F. Bernardo, M. Oliveira

**Affiliations:** ^1^Interdisciplinary Centre of Research in Animal Health (CIISA), Faculdade de Medicina Veterinária da Universidade de Lisboa, Avenida da Universidade Técnica, 1300-477 Lisboa, Portugal; ^2^ISPA - Instituto Universitário das Ciências Psicológicas, Sociais e da Vida, Rua Jardim do Tabaco 34, 1149-041 Lisboa, Portugal; ^3^National Reference Laboratory of Gastrointestinal Infections, Centro Nacional de Salmonella, National Health Institute Doutor Ricardo Jorge, Avenida Padre Cruz, 1649-016 Lisboa, Portugal

## Abstract

*Salmonella* Typhimurium 1,4,[5],12:i:- is a major serovar responsible for human salmonellosis whose biofilm-forming ability, influenced by environmental conditions like those found in the gastrointestinal tract, is one of the main contributing factors to its ability to persist in the host and thus one of the main causes of chronic relapsing infections. Most studies to evaluate biofilm formation are performed in microtiter assays using standard media. However, no reports are available on the ability of this serovar to produce biofilm under *in vitro* simulated gastrointestinal conditions which better correlate with the environment found in the gastrointestinal tract. To address this, a modified biofilm assay simulating intestinal fluid was conceived to assess the biofilm formation of 133 *Salmonella* Typhimurium 1,4,[5],12:i:- isolates with and without agitation and at three different time points (24 h, 48 h, and 72 h). The results were then compared to the existing microtiter method using conventional biofilm growth medium (Mueller Hinton Broth). Statistical analysis revealed significant differences in the results obtained between the three protocols used. The simulated human intestinal environment impaired biofilm production demonstrating that conditions like pH, agitation or the presence of enzymes can influence biofilm production. Therefore, results from *in vitro* simulation of *in vivo* conditions may contribute to unravelling factors relating to biofilm formation and persistence in the context of the human host.

## 1. Introduction

The emergence of a pandemic monophasic variant of* Salmonella* Typhimurium,* S. enterica* subsp.* enterica* serovar 1,4,[5],12:i:-, was first reported in Europe in the mid-1990s and is presently considered to be one of the major serovars responsible for human salmonellosis worldwide [1].

Many studies have demonstrated that* Salmonella* bacteria are capable of forming biofilms on a wide variety of abiotic and biotic surfaces [2, 3]. These highly organized multicellular bacterial structures, responsible for chronic or persistent infections, decrease antimicrobial therapy efficacy and improve resistance to environmental stresses such as desiccation, high temperatures, and antiseptics [4, 5].

Since its conception by Christensen and collaborators in 1985, the 96-well microtiter plate test has been the most frequently used assay for high throughput quantitative evaluation of biofilm-forming ability by bacteria [6, 7]. Over the years, modifications have been made to improve its accuracy [8, 9]. It is generally performed under static conditions using different media, such as Mueller Hinton Broth (MHB) or Tryptic Soy Broth (TSB), and enables quantitative biofilm determination through the application of different dyes such as crystal violet, resazurin, or dimethyl methylene blue [7, 8].

However these* in vitro* conditions differ greatly from the human intestinal environment, in terms of organic composition (enzymes), pH, or dynamics (peristalsis), which is the preferential location for* Salmonella* infection.

Several factors, including pH, temperature, and media composition [10, 11], affect biofilm formation. We aimed to evaluate the influence of conditions mimicking the intestinal human tract environment on biofilm formation by* Salmonella* Typhimurium 1,4,[5],12:i:-* in vitro*. With these modifications, which better simulate real conditions, we aim to provide a better insight into the influence the gastrointestinal environment has upon the biofilm-forming ability of this serovar and ultimately provide more reliable laboratory and clinically relevant results.

## 2. Materials and Methods

### 2.1. Bacterial Isolates and Identification

In this study, 133* Salmonella* Typhimurium 1,4,[5],12:i:- isolates, collected in Portugal from 2006 to 2011 from different origins, were used. Isolates were obtained from clinical (*n* = 125), environmental (*n* = 5), and animal (*n* = 3) samples. All* Salmonella* isolates were serotyped and identification was confirmed by multiplex PCR as recommended by EFSA (EFSA Panel on Biological Hazards 2010).

### 2.2. Evaluation of Biofilm Formation by a Standard Microtiter Biofilm Assay

Alamar Blue (AB) (Thermo Fisher Scientific, Oxford, UK) biofilm assay was performed according to the protocol described by Pettit et al. (2005) [8], with minor modifications. Overnight cultures were used to prepare bacterial suspensions with 5 × 10^5^ CFU/mL in MHB (Liofilchem, Roseto degli Abruzzi, Italy). Suspensions were placed in flat-bottom, polystyrene, tissue-culture-treated 96-well microtiter plates (Orange Scientific, Braine-l'Alleud, Belgium). Three microtiter wells were used per isolate. Plates were incubated in a humidity chamber at 37°C without agitation for 24 h, 48 h, and 72 h. After each time point, plates were removed from the incubator and 5 *μ*L of AB was added to the wells, gently shaken, and incubated for 1 h at 37°C, in order to stain the adherent and viable bacteria. Absorbances at 570 nm were determined using a Spectra MAX 340PC microplate reader (Molecular Devices, Sintra, Portugal). All microtiter assays were carried out in triplicate and repeated on three different occasions and the results were averaged.

### 2.3. Biofilm Formation under* In Vitro* Simulated Intestinal Conditions by a Microtiter Biofilm Assay

#### 2.3.1. *In Vitro* Passage of* Salmonella* Typhimurium 1,4,[5],12:i:- under Simulated Gastric Conditions

Microtiter biofilm assay was also performed using simulated gastrointestinal conditions as described by de Angelis et al. (2006) [12]. Briefly, stationary-phase bacteria grown in 5 mL of TSB were harvested at 6000 g (Hermle Labortechnik, Wehingen, Germany) for 10 min and suspended in 5 mL of simulated gastric fluid which contained NaCl (125 mM/L), KCl (7 mM/L), NaHCO (45 mM/L), and pepsin (3 g/L) (Sigma-Aldrich, St. Louis, USA), pH 3. Bacterial suspensions were submitted to agitation conditions for 180 min with a minishaker apparatus (VWR, Lisboa, Portugal) at 175 rpm, in order to simulate the passage through the stomach. Aliquots were taken in order to determine the number of colony forming units per mL by measuring optical density (O.D.) values, based on standard curves previously determined (data not shown).

#### 2.3.2. *In Vitro* Biofilm Formation under Simulated Intestinal Conditions

After gastric digestion, bacteria cells were harvested using the same conditions, washed with 0.9% sterile sodium chloride solution, and suspended in simulated intestinal fluid (SIF), containing 0.1% (w/v) pancreatin (AppliChem, Darmstadt, Germany) and 0.15% (w/v) bile bovine (Sigma-Aldrich, St. Louis, USA), pH 8.0 [12].

Then, 100 *μ*L of bacterial suspensions in SIF was incubated in flat-bottom, polystyrene, and tissue-culture-treated 96-well microtiter plates (Orange Scientific, Braine-l'Alleud, Belgium). For each isolate, three microtiter wells were used. Plates were incubated in a humidity chamber at 37°C under stationary and agitation conditions with a minishaker apparatus (VWR, Lisboa, Portugal) at 100 rpm for 24, 48, and 72 h, and, after each time point, plates were removed from the incubator and 5 *μ*L of AB was added to the wells. The plates were then incubated for a further 1 h at 37°C. Absorbances at 570 nm were determined using a Spectra MAX 340PC microplate reader (Molecular Devices, Sintra, Portugal). All microtiter assays were carried out in triplicate and repeated on three different occasions and the results were averaged.

#### 2.3.3. Classification of Biofilm-Forming Ability by Microtiter Plates

Based on the O.D. and O.D. cut-off (O.D.c) values, isolates were classified into different categories according to their biofilm-forming ability, as previously described by Stepanovic et al. (2000) [9]. The O.D. cut-off was defined as three standard deviations above the mean O.D. of the negative control and isolates were classified as follows: if O.D. ⩽ O.D.c, isolates were considered to be nonbiofilm producers; if O.D.⩽2 × O.D.c, isolates were considered weak biofilm producers; if 2 × O.D.c < O.D.⩽4 × O.D.c, isolates were considered moderate biofilm producers; and if 4 × O.D.c < O.D., isolates were considered strong biofilm producers [13]. AB assays were performed in triplicate and repeated on different occasions, and results were averaged. Results are presented as mean value ± standard deviation (SD). Statistical analyses were performed using the SPSS 20.0 software (IBM Corporation, NY, USA). Differences between time points and techniques were evaluated by repeated measures ANOVA and one-way ANOVA, respectively. Tukey post hoc tests were used to compare biofilms O.D. mean values. Correlation between CFU/mL after gastric passage and biofilm production at 24 h was determined by Pearson coefficient. *P* values ⩽ 0.05 were considered statistically significant.

## 3. Results and Discussion

**Figure 1:**
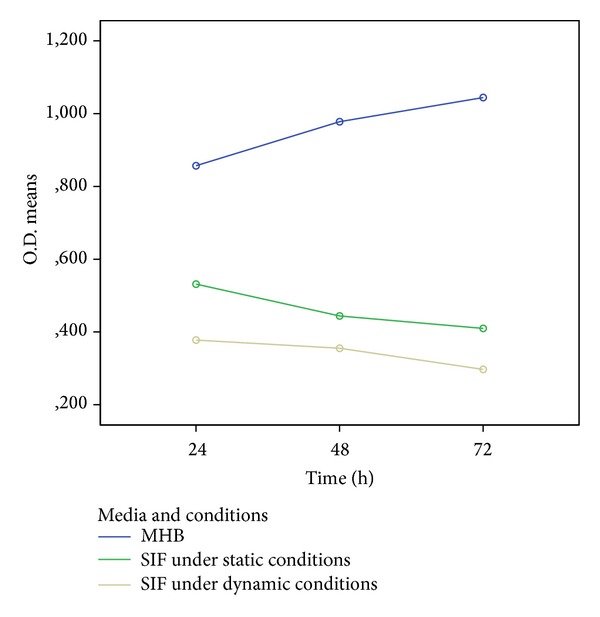
Time course of biofilm production by 133* Salmonella* Typhimurium 1,4,[5],12:i:- isolates using an Alamar Blue microtiter assay applied in different incubation conditions. Mean and standard deviation for MHB were at 24 h 0,856 ± 0,095, at 48 h 0,977 ± 0,105, and at 72 h 1,044 ± 0,118. SIF under static conditions were at 24 h 0,531 ± 0,217, at 48 h 0,443 ± 0,222, and at 72 h 0,409 ± 0,146. SIF under agitation conditions were at 24 h 0,377 ± 0,136, at 48 h 0,355 ± 0,142, and at 72 h 0,297 ± 0,108. MHB: Mueller Hinton Broth; SIF: simulated intestinal fluid.

Standard microtiter biofilm assay staining with resazurin (Alamar Blue), a metabolic activity indicator frequently used for quantitative biofilm determination, revealed that* Salmonella* Typhimurium 1,4,[5],12:i:- isolates possess a high ability for biofilm formation on plastic surfaces, which is in accordance with previous studies [7, 10, 14]. O.D. mean values in MHB increased over time; it was observed that biofilms with the highest O.D. mean values are produced at 72 h (Figure 1). This increase was statistically significant (repeated measures ANOVA, *P* ≤ 0.001).

Following the simulated gastric passage using the modified microtiter biofilm assay, CFU/mL values have a significant positive correlation, although weak, with biofilm production at 24 h in SIF under static conditions (Pearson *r* = 0,183, *P* = 0.018) and in SIF under dynamic conditions (Pearson *r* = 0,158, *P* = 0.035). Higher numbers of CFU/mL can lead to a higher biofilm formation, even though the effects of gastric stress conditions on biofilm formation may be strain specific, as demonstrated by other authors [15].

The largest number of isolates forming weak biofilms was found in SIF under dynamic conditions (83.5% at 24 h, 51.1% at 48 h, and 57.9% at 72 h), while the largest number of isolates able to form moderate and strong biofilms was found in MHB at 48 h and at 72 h (66.2% and 99.2%, resp.) (Table 1). However, 21% of the isolates showed strong biofilm-forming ability at 24 h in SIF under static conditions, and this percentage decreased with time (9% at 48 h and 3% at 72 h). In MHB, more than one-third (37.6%) of the* Salmonella* Typhimurium 1,4,[5],12:i:- isolatestested were only able to produce strong biofilms at 72 h.

Human gastrointestinal conditions may decrease bacteria's ability to adhere to a substratum, the first step required for biofilm formation and which impaired the ability to form strong biofilm [16]. O.D. mean values of biofilm production in SIF under dynamic conditions decreased significantly with incubation time (repeated measures ANOVA, *P* ≤ 0.001) and are significantly lower in comparison with static conditions (ANOVA, *P* ≤ 0.001), at all the time points studied. This can be explained by the fact that the dynamic conditions applied may have impaired bacterial adhesion and are in accordance with other reports that used dynamic methodologies [11, 16].

Biofilm O.D. mean values obtained in SIF with static conditions, although lower than the ones from MHB, are higher than the ones obtained in SIF with dynamic conditions; these differences are statistically significant (ANOVA, *P* ≤ 0.001) showing that conditions, like agitation, have a significant influence on biofilm formation. Dynamics of intestinal peristalsis may strongly influence bacteria's ability to adhere to a surface and should be included as a parameter during biofilm evaluation, as already stated in previous studies [16].

The decrease in biofilm OD mean values between 48 h and 72 h at SIF with dynamic condition was significantly higher than in SIF under static conditions (Tukey, *P* ≤ 0.001), which can be due to a decrease in the number of viable bacteria. The higher number of dead bacteria cells may be due to a decrease in nutrients together with an accumulation of toxic compounds originating from bacterial metabolism that were disseminated by the agitation conditions during this assay [17].

There were significant differences between results obtained by the three protocols at the three time points evaluated (ANOVA, *P* ≤ 0.001), which indicates that intestinal conditions can influence biofilm production by* Salmonella*. White et al. 2008 [18] previously showed that expression of biofilm related genes like curli genes is turned off during* in vivo* infection but turned on again once the bacteria is shed into the environment. This may explain why biofilm production is lower in SIF than in MHB, especially if considering the dynamic conditions present in the intestinal tract due to peristalsis.

## 4. Conclusions

The simulated gastrointestinal environment impaired biofilm production by* Salmonella*, demonstrating that conditions simulating those encountered* in vivo* like pH, agitation, or the presence of enzymes can influence* in vitro* biofilm formation results, emphasizing the importance of experimental conditions in the results obtained. In conclusion, the provision of dynamic and environmental conditions that better simulate the* in vivo* gastrointestinal stress that* Salmonella* is subjected to should be included as one of the parameters in the evaluation of biofilm producing strains, enabling a more accurate correlation between* in vitro* biofilm formation and what happens in the gastrointestinal tract. By approximating experimental conditions to those that bacteria encounter in the human host it may be possible to obtain more insight into the real ability and importance of biofilm production when compared with MHB used in standard biofilm assays.

## Figures and Tables

**Table 1:** Characterization of biofilm-forming ability of 133 *Salmonella* Typhimurium 1,4,[5],12:i:- isolates using an Alamar Blue microtiter assay applied in different incubation conditions.

Biofilm formation ability	Number of strains that produced biofilm (*n*, %)								
	24 h			48 h			72 h		
	MHB	SIF with static conditions	SIF with dynamic conditions	MHB	SIF with static conditions	SIF with dynamic conditions	MHB	SIF with static conditions	SIF with dynamic conditions
Strong biofilm producer	0.0%	21.8% (*n* = 29)	0.8% (*n* = 1)	0.0% (*n* = 0)	9.0% (*n* = 12)	0.8% (*n* = 1)	37.6% (*n* = 50)	6.8% (*n* = 9)	3.0% (*n* = 4)
Moderate biofilm producer	54.1% (*n* = 72)	42.1% (*n* = 46)	15.8% (*n* = 21)	66.2% (*n* = 88)	39.8% (*n* = 53)	37.6% (*n* = 50)	61.7% (*n* = 82)	52.6% (*n* = 70)	39.1% (*n* = 52)
Weak biofilm producer	45.9% (*n* = 61)	36.1% (*n* = 58)	83.5% (*n* = 111)	33.8% (*n* = 45)	51.1% (*n* = 68)	61.7% (*n* = 82)	0.8% (*n* = 1)	40.6% (*n* = 54)	57.9% (*n* = 77)

MHB: Mueller Hinton Broth; SIF: simulated intestinal fluid.

## References

[1] Switt AIM, Soyer Y, Warnick LD, Wiedmann M (2009). Emergence, distribution, and molecular and phenotypic characteristics of *Salmonella enterica* serotype 4,5,12:i:-. *Foodborne Pathogens and Disease*.

[2] Møretrø T, Vestby LK, Nesse LL, Storheim SE, Kotlarz K, Langsrud S (2009). Evaluation of efficacy of disinfectants against Salmonella from the feed industry. *Journal of Applied Microbiology*.

[3] Ledeboer NA, Jones BD (2005). Exopolysaccharide sugars contribute to biofilm formation by *Salmonella enterica* serovar typhimurium on HEp-2 cells and chicken intestinal epithelium. *Journal of Bacteriology*.

[4] Scher K, Romling U, Yaron S (2005). Effect of heat, acidification, and chlorination on *Salmonella enterica* serovar typhimurium cells in a biofilm formed at the air-liquid interface. *Applied and Environmental Microbiology*.

[5] Marin C, Hernandiz A, Lainez M (2009). Biofilm development capacity of *Salmonella* strains isolated in poultry risk factors and their resistance against disinfectants. *Poultry Science*.

[6] Peeters E, Nelis HJ, Coenye T (2008). Comparison of multiple methods for quantification of microbial biofilms grown in microtiter plates. *Journal of Microbiological Methods*.

[7] Stepanović S, Cirković I, Ranin L, Svabić-Vlahović M (2004). Biofilm formation by *Salmonella* spp. and *Listeria monocytogenes* on plastic surface. *Letters in Applied Microbiology*.

[8] Pettit RK, Weber CA, Kean MJ (2005). Microplate alamar blue assay for *Staphylococcus epidermidis* biofilm susceptibility testing. *Antimicrobial Agents and Chemotherapy*.

[9] Stepanovic S, Vukovic D, Dakic I, Savic B (2000). Svabic-Vlahovic M: a modified microtiter-plate test for quantification of staphylococcal biofilm formation. *Journal of Microbiological Methods*.

[10] Solomon EB, Niemira BA, Sapers GM, Annous BA (2005). Biofilm formation, cellulose production, and curli biosynthesis by *Salmonella* originating from produce, animal, and clinical sources. *Journal of Food Protection*.

[11] Stepanović S, Cirkovic I, Mijac V, Svabic-Vlahovic M (2003). Influence of the incubation temperature, atmosphere and dynamic conditions on biofilm formation by *Salmonella* spp. *Food Microbiology*.

[12] de Angelis M, Siragusa S, Berloco M (2006). Selection of potential probiotic lactobacilli from pig feces to be used as additives in pelleted feeding. *Research in Microbiology*.

[13] Stepanović S, Cirković I, Ranin L, Svabić-Vlahović M (2004). Biofilm formation by *Salmonella* spp. and *Listeria monocytogenes* on plastic surface. *Letters in Applied Microbiology*.

[14] Vestby LK, Møretrø T, Langsrud S, Heir E, Nesse LL (2009). Biofilm forming abilities of *Salmonella* are correlated with persistence in fish meal- and feed factories. *BMC Veterinary Research*.

[15] Lianou A, Koutsoumanis KP (2012). Strain variability of the biofilm-forming ability of *Salmonella enterica* under various environmental conditions. *International Journal of Food Microbiology*.

[16] Stepanović S, Vuković D, Jezek P, Pavlović M, Svabic-Vlahović M (2001). Influence of dynamic conditions on biofilm formation by staphylococci. *European Journal of Clinical Microbiology & Infectious Diseases*.

[17] Sillankorva S, Neubauer P, Azeredo J (2008). *Pseudomonas fluorescens* biofilms subjected to phage phiIBB-PF7A. *BMC Biotechnology*.

[18] White AP, Gibson DL, Grassl GA (2008). Aggregation via the red, dry, and rough morphotype is not a virulence adaptation in *Salmonella enterica* serovar typhimurium. *Infection and Immunity*.

